# Sex, Age, and Ethnic Background Shape Adaptive Immune Responses Induced by the SARS-CoV-2 mRNA Vaccine

**DOI:** 10.3389/fimmu.2022.786586

**Published:** 2022-03-28

**Authors:** Jie Bai, Asako Chiba, Goh Murayama, Taiga Kuga, Naoto Tamura, Sachiko Miyake

**Affiliations:** ^1^ Department of Immunology, Juntendo University Graduate School of Medicine, Tokyo, Japan; ^2^ Department of Internal Medicine and Rheumatology, Juntendo University School of Medicine, Tokyo, Japan

**Keywords:** SARS-CoV-2, mRNA vaccine, antigen-specific T cell response, T peripheral helper cell, SARS-CoV-2 variants of concern, HLA

## Abstract

Severe acute respiratory syndrome coronavirus 2 (SARS-CoV-2) mRNA vaccine-induced adaptive responses have been well investigated. However, the effects of sex, age, and ethnic background on the immune responses elicited by the mRNA vaccine remain unclear. Here, we performed comprehensive analyses of adaptive immune responses elicited by the SARS-CoV-2 mRNA vaccine. Vaccine-induced antibody and T cell responses declined over time but persisted after 3 months, and switched memory B cells were even increased. Spike-specific CD4^+^ T and CD8^+^ T cell responses were decreased against the B.1.351 variant, but not against B.1.1.7. Interestingly, T cell reactivity against B.1.617.1 and B.1.617.2 variants was decreased in individuals carrying HLA-A24, suggesting adaptive immune responses against variants are influenced by different HLA haplotypes. T follicular helper cell responses declined with increasing age in both sexes, but age-related decreases in antibody levels were observed only in males, and this was associated with the decline of T peripheral helper cell responses. In contrast, vaccine-induced CD8^+^ T cell responses were enhanced in older males. Taken together, these findings highlight that significant differences in the reactogenicity of the adaptive immune system elicited by mRNA vaccine were related to factors including sex, age, and ethnic background.

## Introduction

T cells and antibodies have critical roles in antiviral immunity against severe acute respiratory syndrome coronavirus 2 (SARS-CoV-2) (1, 2). To enter host cells, SARS-CoV-2 interacts with the angiotensin-converting enzyme 2 receptor expressed on host cells *via* their receptor-binding domain (RBD) in the S1 subunit of the spike protein. Thus, neutralizing antibodies that bind to the spike RBD have a critical role in antiviral immunity by blocking the entry of SARS-CoV-2 into the host cells. Indeed, a cocktail of neutralizing antibodies that target the RBD was shown to be useful in the treatment of coronavirus disease 2019 (COVID-19) (3). T cells also have protective roles in antiviral immunity (4, 5). T follicular helper (Tfh) cells provide cognate help to B cells to differentiate into antibody producing cells. Tfh cell responses were observed in COVID-19, and numbers of Tfh cells and RBD-specific memory B cells were associated with viral-specific antibody production (6, 7). CD8^+^ T cells kill infected cells, and T helper 1 (Th1) cells exert antiviral immunity by the production of cytokines including interferon-γ (IFN-γ) (1). Several reports indicated that strong T cell responses were associated with milder COVID-19 (4, 5, 8–10), and that poor T cell responses were associated with disease severity in male patients (11). In patients with impaired B cell function caused by hematologic cancer, including those receiving anti-CD20 therapy, T cell responses were important for the improved outcome of COVID-19 (12, 13). Thus, the induction of adequate T and B cell responses by vaccination is desired for protection against SARS-CoV-2 infection.

Two SARS-CoV-2 mRNA-based vaccines that encode the spike glycoprotein and demonstrated protective efficacy have been used globally (14–17). SARS-CoV-2 mRNA vaccines elicited robust antibody production after booster vaccination (18–21). The strong responses of T cells including Th1 and CD8^+^ T cells were also observed after booster vaccination (19, 22–27). Several important questions need to be addressed to better understand the effects of these new mRNA vaccines on the adaptive immune response. These include how long the immunological memory against SARS-CoV-2 persists, and how age, sex, and ethical differences influence the adaptive immune responses elicited by the vaccine. Previous studies reported that serum levels of SARS-CoV-2 spike-binding or neutralizing antibodies showed a relatively modest reduction at 3 months compared with at 1 month (28–30). Spike-specific T cell responses were also high after 1 month and were decreased after 3 months, although spike-specific T cells were present at higher numbers than before vaccination (19). Recently, high frequencies of spike-binding germinal center B cells and plasmablasts were demonstrated to be present in lymph nodes 12 weeks after booster immunization (31). Regarding the influence of age on vaccine-induced immune responses, antibody responses were decreased with increasing age, except for one study showing no influence of age on antibody levels (18, 21, 32–35). mRNA vaccine-induced T cell responses were also shown to be reduced in older adults (33). Most of these studies focused on specific immune responses such as antibody or T cell responses, and the characteristics of subjects were different between studies. Thus, a more comprehensive analysis of adaptive immune responses is desired.

During the SARS-CoV-2 pandemic, various mutants have emerged (36). Thus, another important question is whether the current SARS-CoV-2 mRNA vaccines elicit adaptive immune responses against SARS-CoV-2 variants of concern (VOCs). Several studies reported neutralizing titers of sera from individuals receiving the SARS-CoV-2 mRNA vaccine were reduced against variants including B.1.1.7, B1.351, and P1 (26, 37–42). However, other studies demonstrated preserved neutralizing titers for B.1.1.7 (42, 43). A recent study showed decreased neutralizing titers for B1.351, and P1, and preserved titers for B.1.1.7 (44). Regarding T cell responses against the variants, reduced reactivity against B.1.351 was observed in some studies, whereas another study demonstrated comparable T cell responses against the B.1.1.7 and B.1.351 variants when compared with wild-type controls (23, 26, 41). The effect of SARS-CoV-2 mRNA vaccine on VOCs appears to vary depending on the study, perhaps because of the different experimental approaches and ethnic backgrounds between the studies.

We obtained sera and peripheral blood mononuclear cells (PBMCs) from individuals before and after vaccination with the BNT162b2 SARS-CoV-2 mRNA vaccine and assessed the humoral and cellular responses. All individuals developed antibody and T cell responses against the SARS-CoV-2 spike protein. Among the SARS-CoV-2 variants, including B.1.1.7, B.1.351, B.1.617.1, and B.1.617.2, SARS-CoV-2 spike-reactive CD4^+^ T and CD8^+^ T cell responses were decreased against the B.1.351 and B.1.617.1 variants. CD8^+^ T cell reactivity against the B.1.617.1 and B.1.617.2 variant antigens was decreased in HLA-A24 carrying individuals, but not in HLA-A24-negative individuals. These results indicate that adaptive immune responses against the SARS-CoV-2 variants are influenced by different HLA haplotypes. Moreover, we revealed that the adaptive immune responses elicited by the mRNA vaccine were greatly influenced by age and sex. Tfh cell responses were decreased with increasing age in the male and female groups. However, the reduction of antibody levels with increasing age was observed only in males, and this was associated with reduced T peripheral helper cell (Tph) responses with increasing age. Thus, the preserved Tph cell responses may contribute to the maintenance of antibody levels in older females. We also revealed that spike-reactive CD8^+^ T cells were increased with increasing age only in males.

## Materials and Methods

### Study Design

Participants were consecutively recruited at Juntendo University. All participants were Asian and received two doses of the BNT162b2 (Pfizer/BioNTech) mRNA vaccine with an interval of 20–27 days. Those who had been diagnosed with COVID-19 and/or were treated with systemic immunosuppressive agents were ineligible for the study. Individuals with serum positive for the anti-SARS-CoV-2 nucleocapsid antibody in NG-Test IgG-IgM COVID-19 (NG Biotech, Guipry, France) were excluded from the study. A total of 89 participants including 62 health care workers who had received the first dose of the BNT162b2 mRNA vaccine between March 13^th^ 2021 and July 3^rd^ 2021, were included in the study. Peripheral blood samples from 62 participants were collected at before, at 1 month and 3 months after the first dose, and 75 participants provided blood samples before and at 1 month and 14 participants provided samples at 1 month after the first dose. The characteristics of the study participants are summarized in **Table 1**.

**Table 1:** Characteristics of the study participants.

	Pre, 1 month, 3 months	1 month	Aim assays (Figure 2A)			
			B.1.1.7	B.1.351	B.1.617.1	B.1.617.2
Total - n	62	89	88	47	27	31
HCW - n (%)	53 (85%)	62 (70%)	62 (70%)	23 (49%)	4 (15%)	5 (16%)
Sex - n (%)						
Male - n (%)	34 (55%)	52 (58%)	51 (58%)	28 (60%)	15 (56%)	19 (61%)
Female - n (%)	28 (45%)	37 (42%)	37 (42%)	19 (40%)	12 (44%)	12 (39%)
Age (years)						
Median	33.0	33.0	33.5	39.0	24.0	24.0
IQR	(29.3–42.5)	(28.0–49.0)	(28.0–49.0)	(23.5–52.0)	(22.0–54.5)	(21.5–53.0)
Range	(20–79)	(20–79)	(20–79)	(20–79)	(20–79)	(20–79)
20–29 - n (%)	16 (26%)	26 (29%)	25 (28%)	20 (43%)	14 (52%)	16 (52%)
30–39 - n (%)	26 (42%)	28 (31%)	28 (32%)	5 (11%)	0 (0%)	0 (0%)
40–49 - n (%)	9 (15%)	14 (16%)	14 (16%)	6 (13%)	3 (11%)	4 (13%)
50–59 - n (%)	8 (13%)	16 (18%)	16 (18%)	11 (23%)	7 (26%)	8 (26%)
≥60 - n (%)	3 (5%)	5 (6%)	5 (6%)	5 (11%)	3 (11%)	3 (10%)
Comorbidities - n (%)						
Hypertension	5 (8%)	8 (9%)	8 (9%)	6 (13%)	4 (15%)	4 (13%)
Diabetes mellitus	1 (2%)	2 (2%)	2 (2%)	2 (4%)	1 (4%)	1 (3%)
Hyperuricemia	4 (6%)	4 (4%)	4 (5%)	0 (0%)	0 (0%)	0 (0%)
Allergic disease	5 (8%)	8 (9%)	8 (9%)	5 (11%)	3 (11%)	3 (10%)
Dyslipidemia	3 (5%)	6 (7%)	6 (7%)	4 (9%)	3 (11%)	3 (10%)
Bronchial asthma	1 (2%)	1 (1%)	1 (1%)	0 (0%)	0 (0%)	0 (0%)
Kidney disease	1 (2%)	1 (1%)	1 (1%)	1 (2%)	0 (0%)	0 (0%)
Neurologic disorder	2 (3%)	2 (2%)	2 (2%)	2 (4%)	1 (4%)	1 (3%)

HCW, health care worker; IQR, interquartile range.

### PBMC and Sera Isolation

PBMCs were isolated from whole blood by density-gradient centrifugation using the BD Vacutainer CP Mononuclear Cell Preparation Tube with Sodium Heparin (BD Biosciences, Franklin Lakes, NJ, USA). PBMCs were immediately used for activation-induced marker (AIM) assays or flow cytometry analysis. Whole blood was collected in 4-ml tubes without anticoagulant and centrifuged at 1800 x g for 15 min, and sera were aliquoted and stored at −80°C.

### Activation-Induced Marker Assay

The following PepTivator SARS-CoV-2 overlapping peptide pools were obtained from Miltenyi Biotec(Bergisch Gladbach, Germany): “S1” (the complete N-terminal S1 domain of the spike glycoprotein including RBD; aa 1–692), “S+” (the sequence domain aa 689–895 in the S2 domain of the spike glycoprotein), “S” (the immunodominant sequence domains of the spike glycoprotein), “M” (the complete sequence of the membrane glycoprotein), “N” (the complete sequence of the nucleocapsid phosphoprotein), mutation pools of B.1.1.7, B.1.351, B.1.617.1, and B.1.617.2, as well as wild-type reference pools for each mutation pool. Peptide pools of CMV pp65 protein and human influenza A (H1N1) virus hemagglutinin were also obtained from Miltenyi Biotec. Each lyophilized peptide pool was reconstituted with sterile distilled water and used for AIM assays following the manufacturer’s instructions. One million PBMCs were cultured in 96-well flat-bottom plates in TexMACS medium (Miltenyi Biotec, catalog number 130-097-196) containing peptide pools (0.6 nmol/mL) at 37°C in a 5% carbon dioxide incubator. PBMCs under the unstimulated condition were cultured in medium only. Twenty-two hours later, cells were incubated with Zombie Yellow Fixable Viability Dye and Fc receptor blocking solution (both from BioLegend, San Diego, CA, USA) and stained with a mixture of monoclonal antibodies against human cell-surface antigens in diluted form: anti-CD3-Brilliant Violet 421(clone UCHT1), anti-CD4-APC/Fire 750 (clone RPA-T4), anti-CD8-PE (clone SK1), anti-OX40-FITC (clone Ber-ACT35), anti-CD137(4-1BB)-APC (clone 4B4-1), and anti-CD69-PE/Dazzle 594 (clone FN50). PBMCs were also stained with anti-HLA-A24-PE (clone 17A10) to distinguish HLA-A24 carriers among subjects. Data were acquired on a FACS LSR Fortessa (BD Biosciences) and analyzed by using FlowJo software (TreeStar, Ashland, OR, USA).

### T and B Cell Subset Analysis by Flow Cytometry

PBMCs were incubated with Fc receptor blocking solution (BioLegend) and then stained with mixtures of the following monoclonal antibodies against human surface antigens: anti-CD3-Brilliant Violet 421(clone UCHT1), anti-CD4-APC/Fire 750 (clone RPA-T4), anti-CD45RA-Brilliant Violet 605 (clone HI100), anti-CD279 (PD1)-PE(clone EH12.2H7), anti-CD185 (CXCR5)-PE/Dazzle594 (clone J252D4), anti-CD38-FITC (clone T16), anti-HLA-DR-V500 (clone G46-6) for T cell subset analysis; anti-CD19-PE/Dazzle594 (clone HIB19), anti-CD20-Alexa Fluor 700 (clone 2H7), anti-CD27-APC/Fire 750 (clone M-T271), anti-IgD-Brilliant Violet 421 (clone IA6-2), anti-CD180-PE (clone G28-8), anti-CD38-FITC (clone T16), anti-HLA-DR-V500 (clone G46-6), and anti-CD138-APC (clone DL-101) for B cell subset analysis. Following surface staining, cells were washed and stained with 7-AAD Viability Staining Solution. Data were acquired on a FACS LSR Fortessa (BD Biosciences) and analyzed by using FlowJo software (TreeStar).

### Measurement of SARS-CoV-2 Spike RBD Antibody

Serum levels of anti-SARS-CoV-2 spike RBD IgG were determined using an ELISA Kit (Acrobiosystems Newark, DE, USA) according to the manufacturer’s instructions.

### Statistical Analysis

All data were analyzed using Graph Pad Prism 9.0 (GraphPad, La Jolla, CA, USA). Differences between groups were analyzed using appropriate tests as indicated in the figure legends.

## Results

### SARS-CoV-2 mRNA Vaccination Induces T Cell Responses Against Spike Antigens

**Figure 1:**
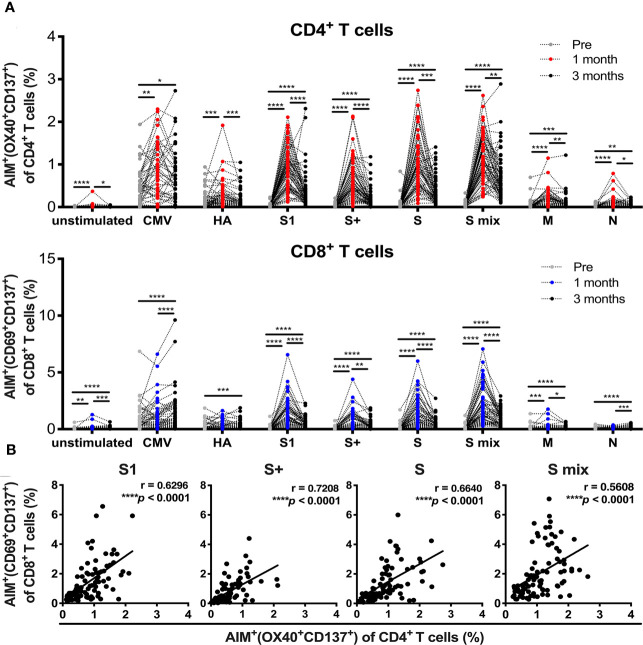
SARS-CoV-2 mRNA vaccination-induced T cell responses. Activation-induced marker (AIM) assays were performed with samples obtained from individuals pre-, and 1 and 3 months post-vaccination. Peripheral blood mononuclear cells were stimulated with peptide pools of SARS-CoV-2 structural protein, cytomegalovirus pp65 protein, and influenza A virus hemagglutinin (HA). Twenty-two hours later, T cells responding to the indicated peptide pools were measured as AIM^+^ (OX40^+^CD137^+^) CD4^+^ T cells and AIM^+^(CD69^+^CD137^+^) CD8^+^ T cells by flow cytometry (FACS). **(A)** Percentages of AIM^+^ (OX40^+^CD137^+^) cells in CD4+ T cells and AIM^+^(CD69^+^CD137^+^) cells in CD8^+^ T cells at pre-, and 1 and 3 months post-vaccination. **(B)** The correlation between AIM^+^ (OX40^+^CD137^+^) CD4^+^ T cells and AIM^+^(CD69^+^CD137^+^) CD8^+^ T cells at 1 month post-vaccination. CMV, cytomegalovirus pp 65 protein; HA, influenza A virus hemagglutinin, S, parts of S1 and S2 domains of SARS-COV-2 spike; S1, SARS-COV-2 spike S1 domain; S+, parts of the S2 domain of SARS-COV-2 spike, S mix, a mixture of S, S1, and S+; M, SARS-COV-2 membrane; N, SARS-COV-2 nucleocapsid. Each dot indicates the value of one individual. **(A)** Statistical differences between groups of pre-, 1 and 3 months were analyzed by the Friedman test. *p < 0.05, **p < 0.01, ***p < 0.001, ****p < 0.0001. **(B)** Correlations were analyzed using Spearman’s correlation analysis.

All participants received two doses of the BNT162b2 SARS-CoV-2 mRNA vaccine (**Table 1**). PBMCs and sera were collected before and 1 and 3 months after the initial administration of the mRNA vaccine. To quantify antigen-specific CD4^+^ T and CD8^+^ T cells, we performed flow cytometry-based activation-induced marker (AIM) assays (2, 45–48). PBMCs were stimulated with 15-mer peptide pools containing 11-amino-acid (aa) overlaps of SARS-CoV-2 structural proteins, comprising “S1” (the complete N-terminal S1 domain of the spike glycoprotein including RBD; aa 1–692), “S+” (the sequence domain aa 689–895 in the S2 domain of the spike glycoprotein), “S” (the immunodominant sequence domains of the spike glycoprotein), “M” (the complete sequence of the membrane glycoprotein), and “N” (the complete sequence of the nucleocapsid phosphoprotein). T cell responses against the peptide pools of cytomegalovirus pp65 protein (CMV) and human influenza A virus hemagglutinin protein (influenza HA) were assessed as controls. Antigen-specific AIM^+^CD4^+^ T and CD8^+^ T cells were identified as “OX40^+^CD137^+^” and “CD69^+^CD137^+^” cells, respectively (**Supplementary Figures 1A, B**). CD4^+^ T and CD8^+^ T cell responses against SARS-CoV-2 spike peptide pools were markedly increased at 1 month after the immunization (**Figure 1A**). The percentages of SARS-CoV-2 spike reactive AIM^+^CD4^+^ T and CD8^+^ T cells were reduced at 3 months post-immunization, but higher than those before immunization (**Figure 1A**). There was a positive correlation between the percentages of AIM^+^CD4^+^ T and CD8^+^ T cells at 1 month post-vaccination (**Figure 1B**). We also observed enhanced responses of CD4^+^T and CD8^+^T cells against antigen pools other than SARS-CoV-2 spike antigens. CD4^+^ T cells reactive to CMV, influenza HA, and SARS-CoV-2 “M” and “N” were increased at 1 month post-immunization. At 3 months post-immunization, “HA”-, “M”-, and “N”-reactive CD4^+^ T cells were decreased, while “CMV”-reactive CD4^+^ T cells were maintained. “M”-reactive CD8^+^ T cells were also increased at 1 month and further increased at 3 months post-immunization. “N”-reactive CD8^+^ T cells were increased at 3 months post-immunization. “CMV”- and “HA”-reactive CD8^+^T cells were most increased at 3 months post-immunization. Interestingly, T cells from individuals who had T cells reactive to “M” or “N” had developed enhanced responses against “CMV” and “HA” after the SARS-CoV-2 mRNA vaccination (**Supplementary Figure 2**).

### SARS-CoV-2 mRNA Vaccination Induces T Cell Responses Against SARS-CoV-2 VOCs

**Figure 2:**
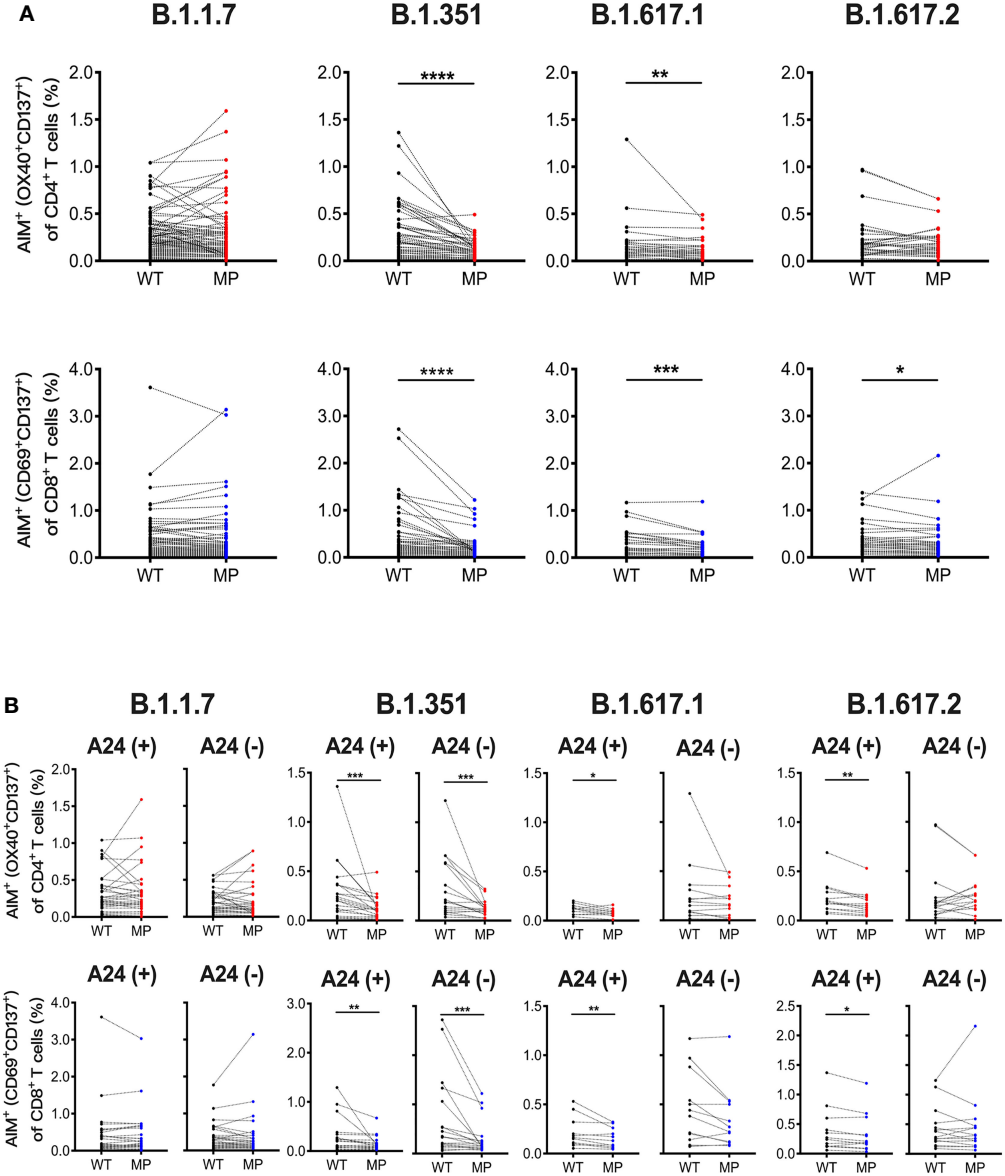
SARS-CoV-2 mRNA vaccination-induced T cell responses against SARS-CoV-2 variants. Peripheral blood mononuclear cells from individuals at 1 month post-vaccination were stimulated for 22 hours with peptide pools that covered the mutated regions (MP) of SARS-CoV-2 and the corresponding wild-type control pools (WT). **(A)** The percentages of AIM^+^ (OX40^+^CD137^+^) CD4^+^ T cells and AIM^+^(CD69^+^CD137^+^) CD8^+^ T cells responding to mutated spike pools (MP) of B.1.1.7 (alpha), B.1.351 (beta), B.1.617.1 (kappa), and B.1.617.2 (delta) as well as each control pool (WT) are shown. **(B)** The percentages of AIM^+^(CD69^+^CD137^+^) CD8^+^ T and AIM^+^ (OX40^+^CD137^+^) CD4^+^ T cells responding to the indicated MP and WT in HLA-A24-positive and -negative individuals are shown. *p < 0.05, **p < 0.01, ***p < 0.001, ****p < 0.0001 (Wilcoxon matched-pairs signed-rank test).

Next, we assessed whether the SARS-CoV-2 mRNA vaccine-induced T cell responses to SARS-CoV-2 variants including B.1.1.7 (alpha), B.1.351 (beta), B.1.617.1 (kappa), and B.1.617.2 (delta) at 1 month post-immunization. AIM assays were performed using overlapping peptide pools covering the mutated regions (MP) and the corresponding wild-type control pools (WT) for each SARS-CoV-2 variant. The percentages of AIM^+^CD4^+^ T and AIM^+^CD8^+^ T cells responding to B.1.351 MP and B.1.617.1 MP, and AIM^+^CD8^+^ T cells reactive to B.1.617.2 MP were lower than those reactive to corresponding WT (**Figure 2A**). The percentages of CD4^+^ T and CD8^+^ T cells reactive B.1.1.7 MP, and B.1.617.2 MP reactive CD4^+^ T cells were comparable to the corresponding controls (**Figure 2A**). The L452R mutation in the spike RBD enhances the viral replication of B.1.617.1 and B.1.617.2 (49). Because L452R is present in the immunodominant epitope presented by HLA-A24, this mutation is thought to enable the virus to escape from HLA-A24-restricted cellular immunity (49). HLA-A24 is a dominant HLA haplotype among Asians including the Japanese population (50). Indeed, approximately half of participants in our study were carrying this HLA haplotype (data not shown). We compared the CD8^+^ T cell reactivity against B.1.617.1 MP and B.1.617.2 MP in HLA-A24-positive and -negative individuals. The percentages of CD8^+^ T cells reactive to these MPs were lower than those reactive to WT in HLA-A24-positive individuals, but such differences were not observed in HLA-A24-negative individuals (**Figure 2B**). The B.1.617.1 MP and B.1.617.2 MP reactive CD4^+^ T cell responses were also decreased in the HLA-A24-positive group, but not in the HLA-A24-negative group (**Figure 2B**). These results indicate that the SARS-CoV-2 mRNA vaccination induces T cell responses to the variants, but that T cell reactivity to some variants is reduced, and that responsiveness to B.1.617 variants is related to HLA haplotype.

### SARS-CoV-2 mRNA Vaccination Elicits T Responses Associated With B Cell Differentiation

**Figure 3:**
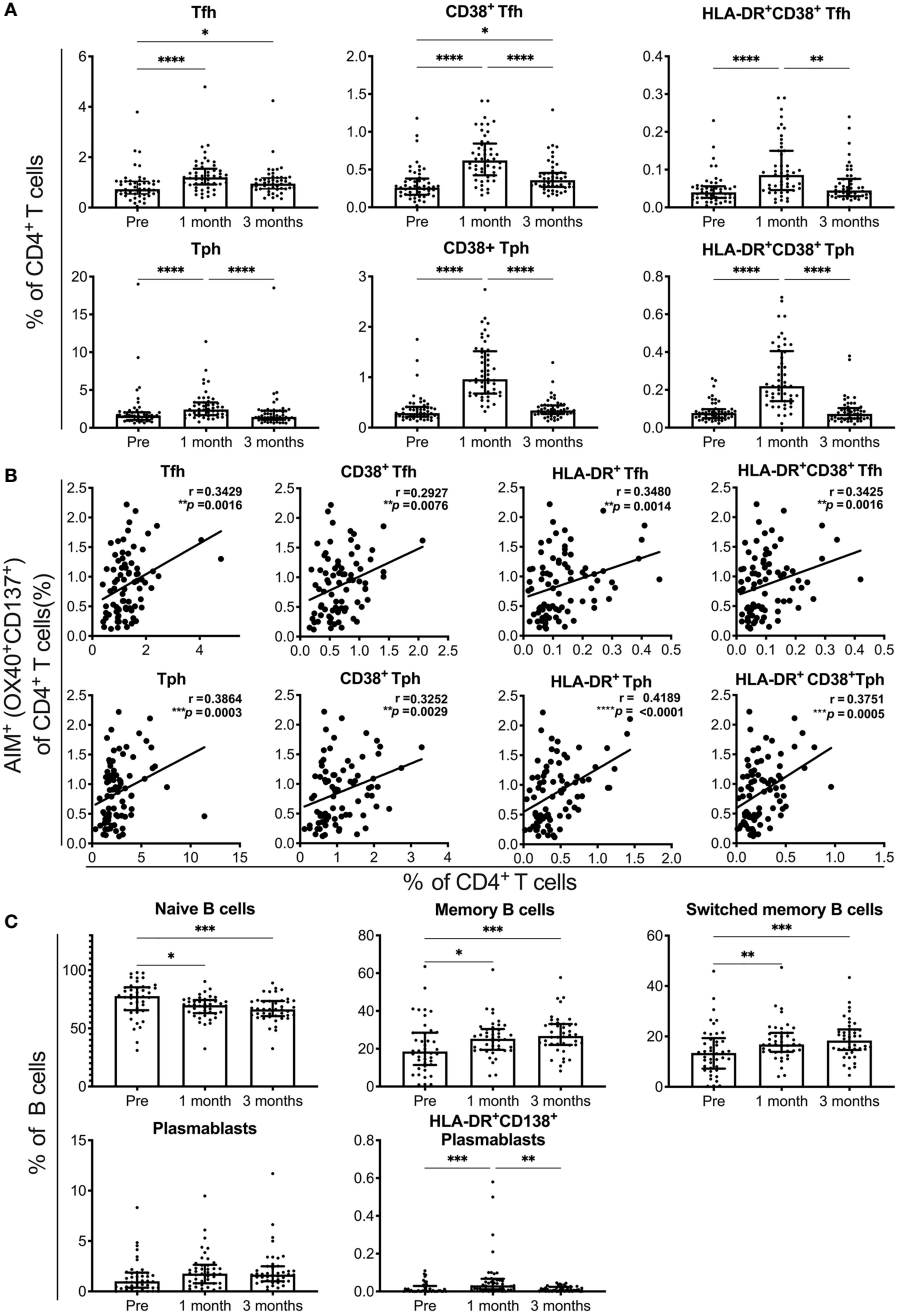
SARS-CoV-2 mRNA vaccination-induced responses of T and B cell subsets. Flow cytometry analysis of peripheral blood mononuclear cells from individuals pre-, and 1 and 3 months post-vaccination. A detailed gating strategy is shown in **Figure S3**. **(A)** The percentages of T follicular helper (Tfh), T peripheral helper (Tph) cells, CD38^+^ and CD38^+^HLA-DR^+^ Tfh, and Tph cells. **(B)** The correlation of AIM^+^CD4^+^ T cells reactive to the S1 peptide pool with total Tfh and Tph cells, and activated Tfh and Tph cells at 1 month post-vaccination. **(C)** The percentages of naïve B, memory B, switched memory B cells, plasmablasts, and HLA-DR^+^CD38^+^ plasmablasts are shown. Each dot indicates the value of one individual. The box indicates the median and the whiskers indicate the first and third quartiles **(A, C)**. **(A, C)** *p < 0.05, **p < 0.01, ***p < 0.001, ****p < 0.0001 (Friedman test). **(B)** Correlations were analyzed using Spearman’s correlation analysis.

We analyzed the responses of Tfh cells, Tph cells, and B cell subsets in individuals who received the SARS-CoV-2 mRNA vaccine (**Supplementary Figure 3**). The percentages of Tfh and Tph cells, as well as activated CD38^+^ cells and CD38^+^ HLA-DR^+^ cells among these cell subsets, were increased at 1 month and reduced at 3 months post-vaccination (**Figure 3A**). The percentages of total and activated Tfh and Tph cells positively correlated with that of AIM^+^CD4^+^ T cells reactive to spike pools at 1 month post-immunization (**Figure 3B** and **Supplementary Figure 4**). Among the B cell subsets, the percentages of naive B cells were decreased and memory B cells, including those that were switched, were increased at 1 and 3 months (**Figure 3C**). Although the frequency of total plasmablasts was unchanged, that of activated HLA-DR^+^CD138^+^ plasmablasts was increased at 1 month post-immunization (**Figure 3C**). These results revealed the different time courses of SARS-CoV-2 mRNA vaccine-induced cellular responses. Strong responses of Tfh and Tph cells, as well as plasmablasts critical for antibody production were observed at 1 month post-vaccination, and memory B cell responses observed at 1 month, continued for 3 months after vaccination.

### The Impact of Age, Sex, and Post-Vaccine Fever on Antibody Responses Against SARS-CoV-2

**Figure 4:**
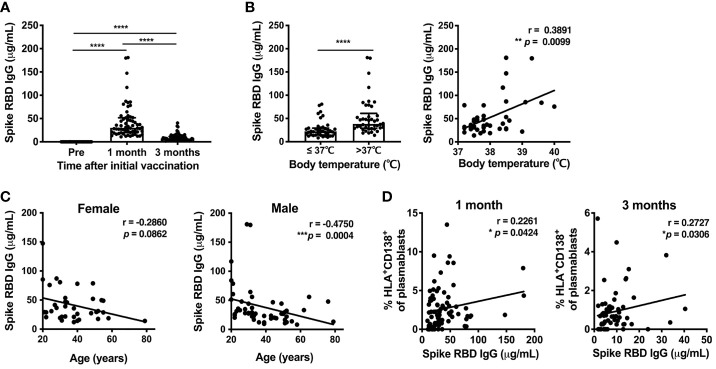
SARS-CoV-2 mRNA vaccination-induced Spike RBD-binding IgG. Serum levels of SARS-CoV-2-Spike RBD-binding IgG were measured by ELISA. **(A)** Serum spike RBD IgG levels at 1 and 3 months after the first dose of SARS-CoV-2 mRNA vaccine. For values below the lower limit of quantification (LLOQ), LLOQ/2 values are plotted. **(B)** The association of serum spike RBD IgG levels at 1 month after vaccination in individuals with post-vaccine fever. Left, comparison of spike RBD IgG levels between individuals with body temperature > or ≤ 37.0°C. Right, correlation between spike RBD IgG levels and body temperature in individuals with body temperature > 37.0°C. **(C)** Correlation between age and serum spike RBD IgG levels at 1 month post-vaccination. **(D)** Correlation between the percentage of HLA-DR^+^CD38^+^ plasmablasts and serum spike RBD IgG levels at 1 month (left) and 3 months (right) after vaccination. Each dot indicates the value of one individual. The box indicates the median and the whiskers indicate the first and third quartiles **(A, B)**. *p<0.05, **p < 0.01, ***p < 0.001, ****p < 0.0001. p-values were determined by Wilcoxon matched-pairs signed-rank test (**A**), and Mann-Whitney U-test (**B**, left). Correlations were analyzed using Spearman’s correlation test **(B–D)**.

To evaluate the antibody response induced by the SARS-CoV-2 mRNA vaccine, the serum levels of spike RBD-binding IgG were measured by ELISA at 1 and 3 months post-vaccination. Serum spike RBD IgG levels showed a robust increase at 1 month (median 29.3μg/mL, IQR 20.61-47.93) and were reduced at 3 months (median 6.8μg/mL, IQR 4.33-10.99) (**Figure 4A**). Anti-spike RBD IgG levels at 1 month post-vaccination were higher in individuals who developed fever after the second dose of the mRNA vaccine, and were positively correlated with body temperature among those that developed fever above 37.0°C (**Figure 4B**). Anti-spike RBD IgG levels were decreased with increasing age in males. Anti-spike RBD IgG levels also tended to be decreased in older females, but the age-related reduction of anti-spike RBD IgG levels was not statistically significant in the female group (**Figure 4C**). To investigate whether B cell responses induced by the vaccination were related to antibody production, we assessed whether anti-spike RBD IgG production was associated with B cell subsets. There was a positive correlation between the percentage of HLA-DR^+^CD138^+^ plasmablasts and the serum spike RBD-binding IgG levels at 1 and 3 months post-immunization (**Figure 4D**).

### Sex and Age Are Related to SARS-CoV-2 T and B Cell Responses After mRNA Vaccination

**Figure 5:**
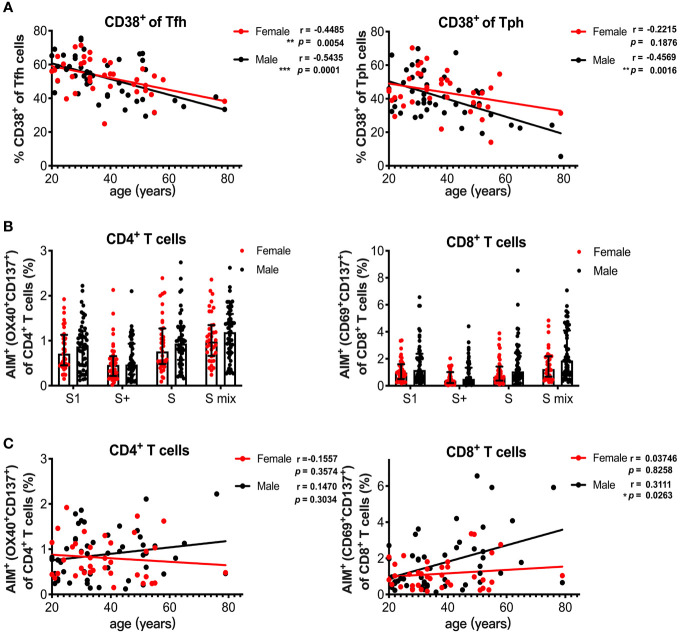
Age and sex influence T and B cell responses elicited by SARS-CoV-2 mRNA vaccination. **(A)** Correlation of age with CD38^+^ Tfh and CD38^+^ Tph cells. **(B)** Comparison of SARS-CoV-2 peptide pool-reactive AIM^+^CD4^+^ T cells and AIM^+^CD8^+^ T cells between female and male individuals at 1 month after vaccination. **(C)** Correlation of age with S1 peptide pool-reactive AIM^+^CD4^+^ T cells and AIM^+^CD8^+^ T cells in male and female groups. Each dot indicates the value of one individual. The box indicates the median and the whiskers indicate the first and third quartiles **(B)**. Correlations were analyzed using Spearman’s correlation test **(A, C)**. *p < 0.05, *p < 0.01, ***p < 0.001.

Sex and age impact immune responses. Indeed, the antibody response induced with BNT162b2 vaccination was reported to be affected by age and sex (32). We also observed a decline in serum spike RBD-binding IgG levels with increasing age in the male group (**Figure 4C**). Because HLA-DR^+^CD138^+^ plasmablasts were associated with spike RBD-binding IgG levels (**Figure 4D**), we analyzed the influence of age and sex on T and B cell responses induced by SARS-CoV-2 mRNA vaccination at 1 month post-immunization. The percentage of CD38^+^ Tfh cells decreased with increasing age in the male and female groups, whereas the percentage of CD38^+^ Tph cells negatively correlated with age only in the male group (**Figure 5A**). We also assessed sex and age differences in the CD4^+^ T and CD8^+^ T cell responses to SARS-CoV-2 peptide pools. We found that CD8^+^ T cells from males tended to have stronger responses to SARS-CoV-2 spike pools than those from females, although there was no significant difference between the groups (**Figure 5B**). Additionally, the percentage of AIM^+^ CD8^+^ T cells from males was increased with increasing age and age positively correlated with AIM^+^ CD8^+^ T cells in the male group, but not with AIM^+^ CD4^+^ T cells in either sex or AIM^+^ CD8^+^ T cells in the female group (**Figure 5C**). Even among individuals at <60 years of age, the percentage of AIM^+^ CD8^+^ T cells tended to be positively correlated with age (r = 0.2689, p = 0.0676) in males, but not in females (r = 0.03139, p = 0.8558). At 3 months post-immunization, CD38^+^Tfh and CD38^+^Tph cells were both decreased with increasing age in in the male and female groups (**Supplementary Figure 5A**). Although the percentages of AIM^+^ CD8^+^ T cells at 3 months in the male group also tended to be higher than those in female groups (**Supplementary Figure 5B**), they did not show a positive correlation with age in males (**Supplementary Figure 5C**).

## Discussion

An increasing number of studies have reported T cell responses elicited by the SARS-CoV-2 mRNA vaccine (19, 22–27, 33, 35, 51–62). Antigen-specific T cell responses are evaluated by using several different methods. Cytokine release assays, namely ELISPOTs or ELISAs are used to determine the responsiveness and the type of cytokines produced by PBMCs stimulated with specific antigens (19, 24, 26, 27, 51–53, 58, 59). AIM assays that stain intracellular cytokines and/or activation markers are also employed to evaluate antigen-specific T cells by using flow cytometry (22, 23, 25, 27, 55–57, 60–62). A benefit of flow cytometry-based AIM assays is that subpopulations of antigen-specific T cells can be characterized. Previous studies using AIM assays have shown that the first vaccine dose induces the spike-specific CD4^+^T cell responses, but the CD8^+^T cell responses become more apparent after the second dose (22). Strong spike-reactive CD4^+^T cells with the capacity to produce IL-2, IFN-γ, and TNF-α are induced by the first dose of the mRNA vaccine, and the second dose of vaccine increases these CD4^+^T cells and induces CD4^+^T cells that produce IL-21 and CD40L (22, 61). However, spike-reactive CD8^+^T cells that produce IFN-γ and TNF-α are observed after the second dose of the vaccine (22, 61).It has been recently demonstrated that 94% and 100% of subjects developed spike-reactive CD4^+^T cells after the first and second doses of vaccine, respectively, and CD8^+^T cell responses were observed in 71% and 88% participants after the first and second doses, respectively (55, 61). However, a recent study observed functional CD8^+^ T cells as early as 6–8 days after the first SARS-CoV-2 vaccine dose, when responsiveness was analyzed at the single-epitope level (25). These functional CD8^+^ T cells were already present when SARS-CoV-2-reactive CD4^+^ T cells and antibodies were barely detectable (25). Because mRNA vaccine-mediated protection has been observed before the second dose when the neutralizing antibody is not yet detectable (17), this early CD8^+^ T cell response might play an important role in protection at the early phase after vaccination. In our study, spike-specific CD4^+^T and CD8^+^T cells developed in all individuals at 1 month after the first dose. Spike-specific CD8^+^T cells have been shown to decline more rapidly than CD4^+^T cells (55). Our study showed that the reduction in AIM^+^cells from 1 month to 3 months post-immunization was comparable between CD4^+^T and CD8^+^T cells. The difference among studies may be due to the differences in experimental systems. Whereas most other studies have been performed using cryopreserved PBMCs, fresh PBMCs were used in our AIM assays. This may have influenced the results because fresh PBMCs respond better than cryopreserved PBMCs. We defined the AIM^+^ cells as OX40^+^CD137^+^ for CD4^+^T cells and CD69^+^OX137^+^ for CD8^+^T cells following the method to identify SRAS-CoV-2 antigen-specific T cells in COVID-19 patients and vaccinated individuals (2, 41). Other studies have used various combinations of surface activation markers, such as CD200, CD40L, CD137, and CD40L, and sometimes with intracellular IFN-γ and TNF-α (23, 25, 55–57, 61, 62). Thus, the different activation markers used to identify activated T cells may have also yielded different results, too.

CD4^+^ T cell responses play major roles in the induction of cellular and humoral immunities. Our study showed that spike-specific CD4^+^T cell responses were positively associated with the responses of Tfh and Tph cells, and spike-specific CD8^+^T cells. CD4^+^ T cell responses elicited by the first dose of vaccine were important for the responses after the booster immunization. Positive correlations have been demonstrated between pre-boost AIM^+^Th1 and AIM^+^Tfh cells with post-boost AIM^+^CD8^+^T cell and neutralizing antibody responses, respectively (61). Thus, it would be important to maintain the levels of spike-specific CD4^+^T cells to maintain the vaccine efficacy and to enhance acquired immune responses after the booster vaccination. In our study, although the numbers of spike-reactive T cells and spike RBD IgG levels were decreased over time, these T cells and antibodies remained present at 3 months post-vaccination. The magnitude of adaptive immune responses after the booster vaccination may influence how long these T cells and antibodies remain in vaccinated individuals. Thus, additional booster vaccinations may be considered for individuals with weak T cell and antibody responses at 1 month post-vaccination. The percentage of switched memory B cells was maintained at high levels at 3 months post-vaccination; however, longitudinal studies are required to determine how long T and B cell memory persists after vaccination.

As previously reported by other groups, a strong response of SARS-CoV-2 spike-reactive antibodies was observed in all individuals after a second dose of the BNT162b2 mRNA vaccine. The serum spike RBD-binding IgG levels were positively associated with the percentage of HLA-DR^+^CD138^+^ plasmablasts and at 1 and 3 months post-immunization, but the correlations were weak. Presumably this is because the activated HLA-DR^+^CD138^+^ plasmablasts post-immunization may include those specific to SARS-COV-2 spike protein that includes the spike RBD. Thus, the analyses of plasmablasts and antibodies both specific to the spike RBD are required to demonstrate their association.

The cut-off values of anti-spike antibodies for protection against COVID-19 are unknown. However, the SRAS-CoV-2 BNT162b2 mRNA vaccine has been shown to induce a strong antibody response especially after the second immunization, to a higher level than that observed in COVID-19 convalescent patients. The first vaccine dose of BNT162b2 induced an anti-spike RBD IgG response in about 88% of individuals, and the RBD IgG levels became positive in 98.4% of individuals and showed a 20-fold increase after the second dose (18). Anti-spike IgG levels after one and two doses of the vaccine were lower in individuals older than 80 years of age than those younger than 80 years of age (33, 63). Other studies have shown that age has a negative effect on anti-spike antibody titers at 14 days after the first dose, but the age-related decline in anti-spike titers is lost after the second immunization or observed only in males (21, 32, 64). Some studies have shown that the anti-spike level decreases with increasing age among individuals younger than 80 years (64, 65). We also observed an age-related decrease in the anti-spike RBD levels only in the male group. Antibody responses after the immunization with the mRNA vaccine are also influenced by other factors, which include immunosuppression, and comorbidities such as diabetes, hypertension, heart disease, and autoimmune diseases (18). Thus, the discrepancies among studies may be due to the subjects in each study.

The bystander activation of CD4^+^ T cells was reported in humans and a mouse model following booster vaccination with tetanus toxoid (66–68). Enhanced responses of CD4^+^ T cells against CMV and influenza HA peptide pools were observed 1 month after SARS-CoV-2 mRNA vaccination. Thus, CMV- and HA-reactive CD4^+^ T cells appeared to be activated by a bystander mechanism after vaccination. The frequencies of CMV- and HA-reactive CD8^+^ T cells tended to be increased at 1 month and were statistically higher at 3 months than those at 1month after SARS-CoV-2 mRNA vaccination. Memory CD8^+^T cells can be activated by cytokines such as IL-12 and IL-18. Thus, AIM^+^CD8^+^ T cells may also include memory CD8^+^T cells activated by cytokine-mediated mechanisms. However, it is not known why the timings of the enhanced responsiveness against non-specific antigens are different between CD4^+^T and CD8^+^T cells. Because the peptide pools used in this study contained epitopes restricted to MHC class I and II, CD4^+^T and CD8^+^T cell responses against class I and class II peptide antigens of control viruses may clarify the differences between CD4^+^T and CD8^+^T cells.

We observed enhanced T cell responses against SARS-CoV-2 membrane and nucleocapsid peptide pools in vaccinated participants. Because these individuals were not diagnosed with COVID-19 and were negative for the anti-SARS-CoV-2 nucleocapsid antibody, it is unlikely that enhanced membrane- and nucleocapsid-reactive T cells were associated with immune responses induced by exposure to SARS-CoV-2. T cell responsiveness against the SARS-CoV-2 membrane and nucleocapsid peptide pools was associated with that against CMV and influenza HA peptide pools after the SARS-CoV-2mRNA vaccination. Enhanced T cell responses against the nucleocapsid have been observed among SARS-CoV-2 infection naïve individuals in other studies (53, 57, 60). The presence of T cells crossreactive with SARS-CoV-2 spike or nucleocapsid antigens in unexposed individuals has been reported (2, 5, 40, 69–71). Thus, bystander activation may have been induced regarding T cells crossreactive with other SARS-CoV-2 structural antigens. However, T cell reactivity against SARS-CoV-2 antigens was observed in both symptomatic and asymptomatic SARS-CoV-2-infected individuals who were negative for anti-nucleocapsid antibodies (4, 9, 72–74). Thus, the possibility that any participant was asymptomatically infected with SARS-CoV-2 during the study and was seronegative for SARS-CoV-2 cannot be excluded.

As described above, the effect of the SARS-CoV-2 mRNA vaccine on the variants appears to vary between studies. This may be because of the different methods used to evaluate antigen-responsive T cells and/or the ethnic groups involved in the studies. In this study, we observed a reduction in the vaccine-induced responses of CD4^+^ T and CD8^+^ T cells against spike peptide pools of the B.1.351 and B.1.617.1 variants, and CD8^+^ T cells reactive against the B.1.617.2 variant. Motozono et al. showed that the L452R mutation of the spike RBD identified in SARS-CoV-2 variants such as B.1.617.1 and B.1.617.2, contributes to viral infectivity by increasing the binding affinity to ACE2, viral replication and fusogenicity. They also found that the L452R mutation is present in an immunodominant epitope presented by HLA-A24 (49), which may evade CD8^+^T cell immunity in individuals who carry HLA-A24. However, it was unknown whether T cell reactivity against B.1.617 mutants elicited by the SRAS-CoV-2 mRNA vaccine was affected by HLA-A24. We demonstrated that the CD8^+^ T cell responses to peptide pools of the B.1.617 variants were decreased in an HLA-A24 dependent manner, but that CD8^+^ T cell responses against the B.1.351 variant were reduced in the HLA-A24-positive and -negative groups. We also observed the decreased reactivity of CD4^+^ T cells to the B.1.617 variants in HLA-A24-positive individuals. This may be related to the indirect effects of reduced spike-reactive CD8^+^ T cells on CD4^+^ T cells, and these reduced CD4^+^ T cell responses may subsequently influence antibody production against the B.1.617 variants. These findings indicate that the HLA haplotype affects the reactivity against certain VOCs and may contribute to the disease course of COVID-19.

The age-related reduction of antibody and T cell responses against SARS-CoV-2 following vaccination with BNT162b2 have been reported (33). In elderly individuals, a lower neutralizing potency of antibodies was accompanied by a reduction in the somatic hypermutation of B cells. In our study, age-related spike RBD IgG reduction was observed in males, and spike RBD IgG levels tended to also be decreased in older females. The frequency of activated Tfh cells in both sexes was decreased with increasing age. Reduced antibody responses were associated with impaired Tfh cell functions in aged mice (75, 76). Thus, the decrease of Tfh cell responses may be at least in part responsible for reduced antibody production associated with aging. Because CD4^+^ T cell responses against spike pools were not influenced by age, the differentiation of Tfh cells may be affected by age. However, the frequency of CD38^+^ Tph cells at 1 month post-vaccination was decreased with increasing age in the male group only. Therefore, Tph cells may contribute to the persistence of antibody responses in older females. Tph cells that promote the differentiation of B cells into antibody producing cells were shown to contribute to antibody production in autoimmune and inflammatory diseases (77–80). However, little is known about the contribution of Tph cells to vaccine-induced antibody responses. Further studies are required to determine whether vaccine-induced Tph cell activation promotes B cell differentiation into antibody producing cells. At 3 months post-vaccination, the frequency of CD38^+^ Tph cells was decreased with increasing age in both sexes. These findings indicate that the induction of Tph cells was not affected by age in females, but age influenced the maintenance of Tph cells in females.

mRNA-based vaccines provoke strong CD8^+^ T cell responses through classical MHC class I antigen presentation (81). Indeed, SARS-CoV-2 mRNA vaccines induce stronger CD8^+^ T cell responses against some spike epitopes compared with natural infection (25). In our study, although spike-reactive CD4^+^ T cell responses were comparable between female and male groups, spike-reactive CD8^+^ T cell responses at 1 month post-vaccination were enhanced and increased with increasing age in males. This tendency was also observed among males younger than 60 years of age. Humoral responses are low in males and elderly individuals, but the effects of age and sex on cellular responses to vaccination are poorly understood. T cell responses induced by influenza virus vaccines are low in elderly individuals (82). Whereas strong CD4^+^ T cell responses to herpes simplex virus peptide vaccines have been observed in females, lymphoproliferative responses to rubella-attenuated vaccines are high in males (82). The reason why spike-reactive CD8^+^ T cell responses were increased with increasing age in males is unclear. In general, females and young adults have stronger innate and adaptive immune responses than males (82–86). Considering higher susceptibility to some viral infections in males, it is possible that there were more SARS-CoV-2-crossreactive CD8^+^ T cells in males who had been infected with the common cold coronavirus. Alternatively, SARS-CoV-2-crossreactive T cells may be maintained more in males. Indeed, at 3 months post-vaccination, more SARS-CoV-2-reactive T cells tended to be present in males of all age groups. In female COVID-19 patients, greater activation and differentiation of CD4^+^ and CD8^+^ T cells has been reported compared with males, although reduced CD8^+^ T cell responses were associated with disease severity in males only (11, 87). Because SARS-CoV-2 spike RBD-binding IgG levels were decreased with increasing age in males, proper CD8^+^ T cell responses may be important for antiviral immunity, especially in older male individuals. As described above, mRNA vaccine-induced CD8^+^ T cells appear to have crucial effects against COVID-19, especially before generating CD4^+^ T cell and antibody responses. However, it is unknown whether these CD8^+^ T cells that increase in males with increasing age are functionally equal to those in younger individuals. Additionally, it is unclear how long these cells have antiviral functions after vaccination. Further studies are needed to assess whether mRNA vaccine-induced CD8^+^ T cells play crucial roles against SARS-CoV-2 infection in larger studies among both sexes and across all age groups.

In summary, we performed a comprehensive analysis of adaptive immune responses elicited by the SARS-CoV-2 mRNA vaccine. We demonstrated that the adaptive immune responses are largely influenced by several factors including, age, sex, and HLA haplotype. These factors may also contribute to the disease course of COVID-19. It is not yet known whether these factors affect the antiviral immunity induced by the mRNA vaccine, but the data presented here may provide important information regarding future vaccine strategies.

## Data Availability Statement

The original contributions presented in the study are included in the article/**Supplementary Material**. Further inquiries can be directed to the corresponding authors.

## Ethics Statement

The studies involving human participants were reviewed and approved by The regional ethics committee at Juntendo University (2020310). The patients/participants provided their written informed consent to participate in this study.

## Author Contributions

AC, NT, and SM designed the research. JB, AC, TK, and GM performed the experiments and data analysis. AC, JB, and SM wrote the manuscript. All authors contributed to the interpretation of the data and preparation of the manuscript. All authors contributed to the article and approved the submitted version.

## Funding

This work was supported by the Japan Society for The Promotion of Science, [Grants-in-Aid for Scientific Research (C)20K08807 to AC, and (B)21H02964 to SM)], a grant from the Institute for Environmental and Gender-specific Medicine, Juntendo University (AC) and a grant from the Japanese Respiratory Foundation (JRF) (AC).

## Conflict of Interest

The authors declare that the research was conducted in the absence of any commercial or financial relationships that could be construed as a potential conflict of interest.

## Publisher’s Note

All claims expressed in this article are solely those of the authors and do not necessarily represent those of their affiliated organizations, or those of the publisher, the editors and the reviewers. Any product that may be evaluated in this article, or claim that may be made by its manufacturer, is not guaranteed or endorsed by the publisher.
