# Supplementary material for: Sex, Age, and Ethnic Background Shape Adaptive Immune Responses Induced by the SARS-CoV-2 mRNA Vaccine
Source: Front Immunol. 2022 Mar 28;13:786586. doi: 10.3389/fimmu.2022.786586 (PMC8995562; doi:10.3389/fimmu.2022.786586)
Supplement: Supplementary file 1 [file DataSheet_1.docx]

**
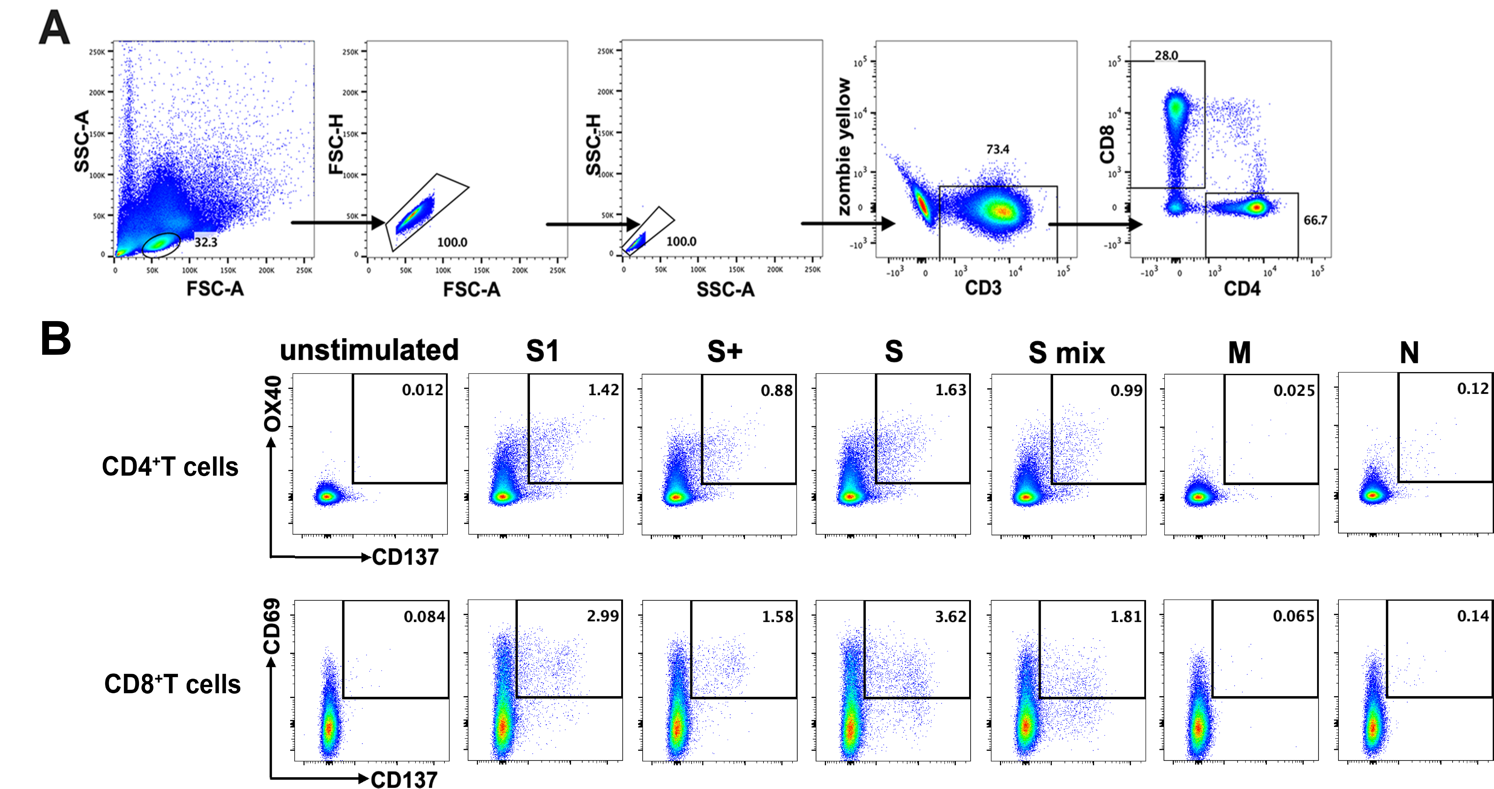
**

**Supplementary Figure 1.** **Gating strategy used for AIM assays**

Activation induced marker (AIM) assays were performed as described in Figure 1. (A) Lymphocytes were gated using a forward scatter area (FSC-A) vs side scatter area (SSC-A) plot. Single cells were selected using FSC-A vs FSC-height (FSC-H) and SSC-A vs SSC-height (SSC-H) plots. Zombie Yellow positive dead cells were excluded. CD4^+^ and CD8^+^ cells among CD3^+^ cells were gated as shown. (B) Representative FACS plots of OX40^+^CD137^+^CD4^+^ T and CD69^+^CD137^+^CD8^+^ T cells at 1 month post-vaccination are shown. S1, SARS-COV-2 spike S1 domain; S+, parts of the S2 domain of SARS-COV-2 spike; S, parts of S1 and S2 domains of SARS-COV-2 spike; S mix, a mixture of S, S1, and S+; M, SARS-COV-2 membrane; N, SARS-COV-2 nucleocapsid.

**
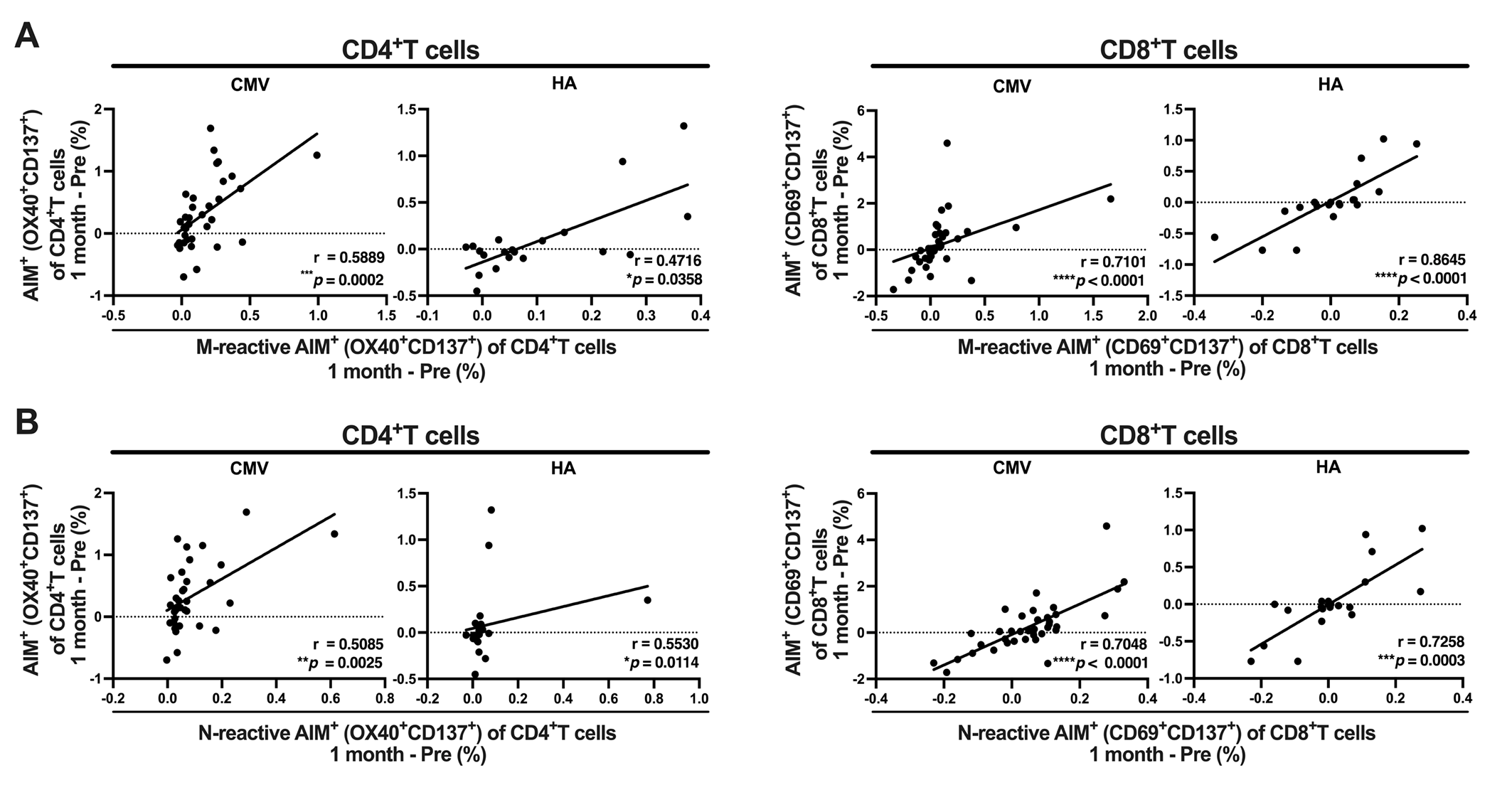
**

**Supplementary Figure 2. T cells reactive to “M” or “N” induce enhanced responses against “CMV” or “HA” after SARS-CoV-2 mRNA vaccination.**

Correlations of the increased percentages of AIM^+^ cells from preimmunization to 1 month after immunization with the SARS-CoV-2 vaccine from experiments in Figure 1 are shown. (A) Correlation between “M”-reactive and “CMV”- or “HA”-reactive CD4^+^ T (left) and CD8^+^ T (right) cells. (B) Correlation between “N”-reactive and “CMV”- or “HA”-reactive CD4^+^ T (left) and CD8^+^ T (right) cells. Each dot indicates the value of one individual. Correlations were analyzed using Spearman’s correlation test. M, SARS-COV-2 membrane; N, SARS-COV-2 nucleocapsid; CMV, cytomegalovirus pp 65 protein; HA, influenza A virus hemagglutinin.


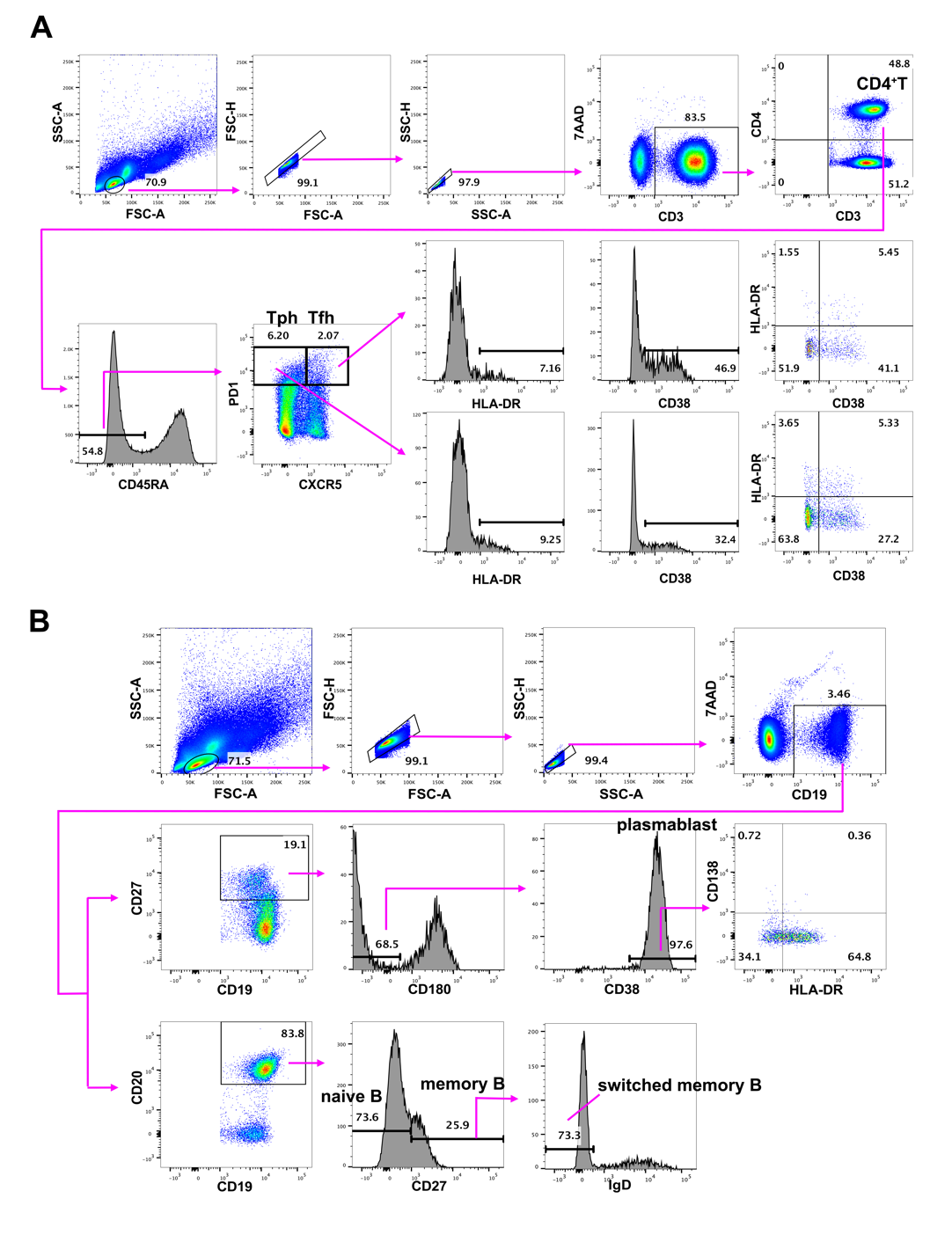


**Supplementary Figure 3.** **Gating strategy for the flow cytometric analysis of T and B cell subsets.** (A) Lymphocytes were gated by using FSC-A vs SSC-A plots, and single cells were selected by using FSC-A vs FSC-H and SSC-A vs SSC-H plots. 7-AAD positive dead cells were excluded. CD3^+^CD4^+^CD45RA^-^ cells were selected and CXCR5^+^PD1^high^ T follicular helper (Tfh) and CXCR5^-^PD1^high^ T peripheral helper (Tph) cells were gated as shown. (B) Lymphocytes and single cells were selected as in (A), and 7-AAD^-^CD19^+^ cells were selected. Among CD19^+^CD20^+^ cells, CD27^+^ memory B cells and IgD^-^CD27^+^ switched memory B cells were gated as shown. Plasmablasts were identified as CD180^-^CD38^+^ cells among CD19^+^CD27^+^ cells, and the expressions of HLA-DR and CD38 were evaluated.

**
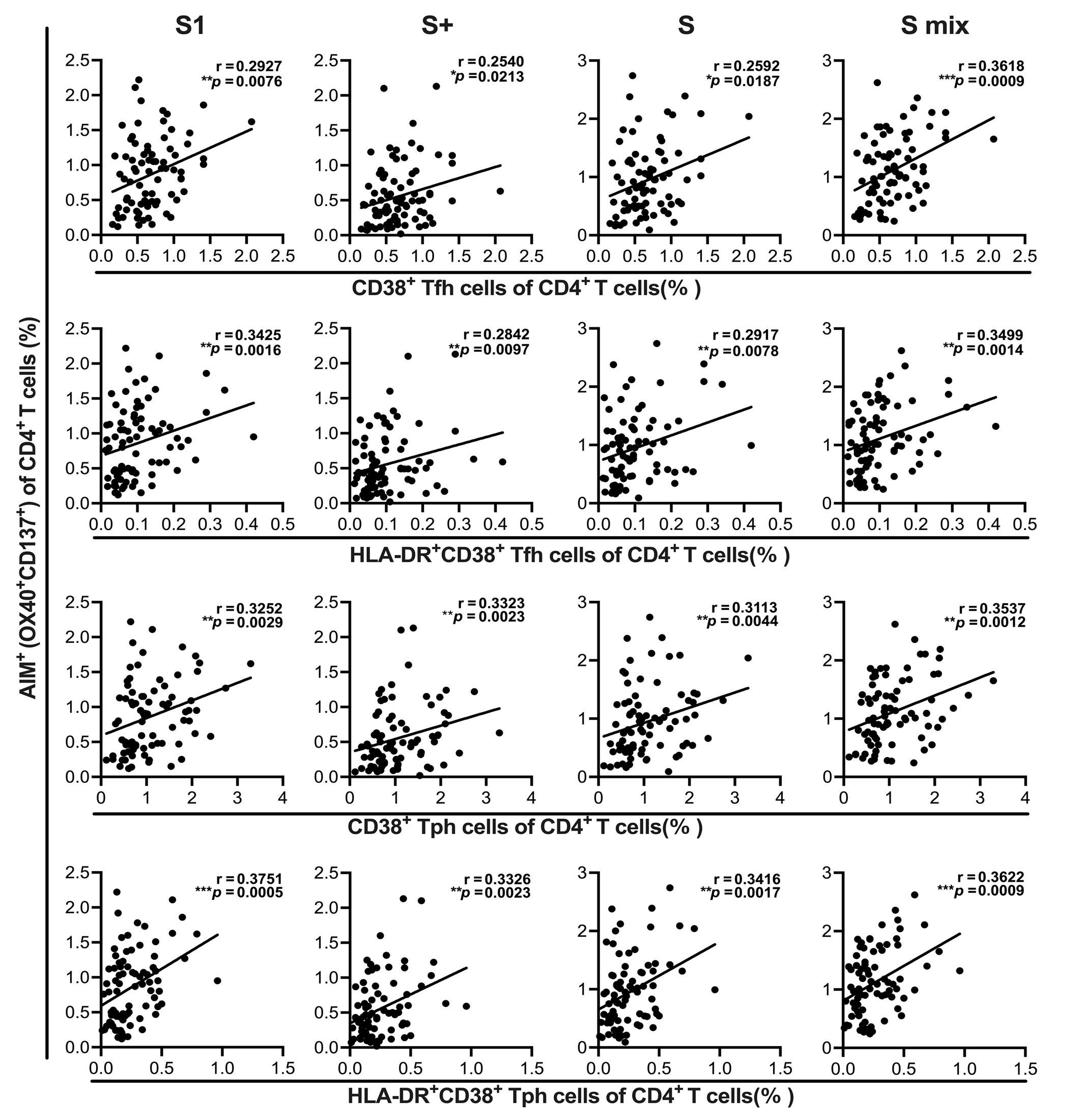
Supplementary Figure 4.** **Correlation of SARS-CoV-2 mRNA vaccination-induced T cell responses with activated Tfh and Tph cells.** The correlation of AIM^+^CD4^+^ T cells reactive to the spike peptide pools with activated Tfh and Tph cells at 1 month post-vaccination. Each dot indicates the value of one individual. Correlations were analyzed using Spearman’s correlation test. S1, SARS-COV-2 spike S1 domain; S+, parts of the S2 domain of SARS-COV-2 spike; S, parts of S1 and S2 domains of SARS-COV-2 spike; S mix, a mixture of S, S1, and S+.

**
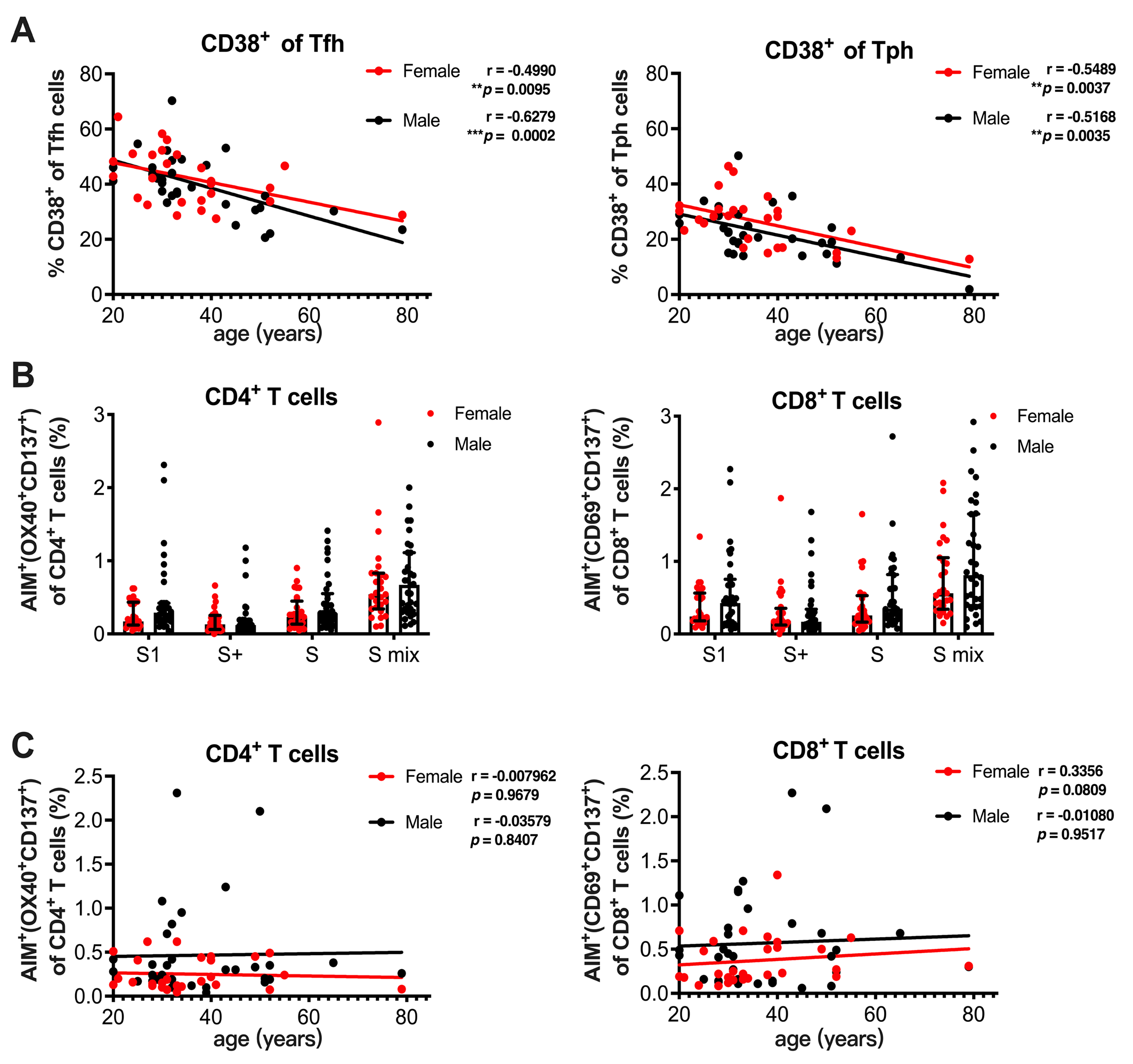
Supplementary Figure 5.** **Age and sex influence T and B cell responses elicited by SARS-CoV-2 mRNA vaccination at 3 months.**

(A) Correlation of age with CD38^+^ Tfh and CD38^+^ Tph cells. (B) Comparison of SARS-CoV-2 peptide pool-reactive AIM^+^CD4^+^ T cells and AIM^+^CD8^+^ T cells between female and male individuals. (C) Correlation of age with S1 peptide pool-reactive AIM^+^CD4^+^ T cells and AIM^+^CD8^+^ T cells in male and female groups. Each dot indicates the value of one individual. The box indicates the median and the whiskers indicate the first and third quartiles (B). Correlations were analyzed using Spearman’s correlation test (A, C).
